# Bipolar radiofrequency ablation of refractory ventricular arrhythmias: results from a multicentre network

**DOI:** 10.1093/europace/euae248

**Published:** 2024-09-27

**Authors:** Piotr Futyma, Arian Sultan, Łukasz Zarębski, Guram Imnadze, Vera Maslova, Stefano Bordignon, Maria Kousta, Sven Knecht, Nikola Pavlović, Petr Peichl, Evgeny Lian, Thomas Kueffer, Daniel Scherr, Michael Pfeffer, Paweł Moskal, Gabriel Cismaru, Bor Antolič, Paweł Wałek, Shaojie Chen, Martin Martinek, Georgios Kollias, Michael Derndorfer, Sebastian Seidl, Boris Schmidt, Jakob Lüker, Daniel Steven, Philipp Sommer, Marek Jastrzębski, Josef Kautzner, Tobias Reichlin, Christian Sticherling, Helmut Pürerfellner, Andres Enriquez, Jonas Wörmann, Julian K R Chun

**Affiliations:** Clinical Electrophysiology, St. Joseph's Heart Rhythm Center, University of Rzeszów, Anny Jagiellonki 17, 35-623 Rzeszów, Poland; Heart Center, Department of Electrophysiology, University Hospital Cologne, Cologne, Germany; Clinical Electrophysiology, St. Joseph's Heart Rhythm Center, University of Rzeszów, Anny Jagiellonki 17, 35-623 Rzeszów, Poland; Clinic for Electrophysiology, Herz- und Diabeteszentrum NRW, Ruhr-Universitaet Bochum, Bad Oeynhausen, Germany; Department of Internal Medicine III (Cardiology and Intensive Care Medicine), University Hospital Schleswig-Holstein (UKSH), Kiel, Germany; Cardioangiologisches Centrum Bethanien, Department Kardiologie, Markus Krankenhaus, Frankfurt, Germany; Department of Internal Medicine II, Cardiology, Angiology, and Intensive Care Medicine, Ordensklinikum Linz Elisabethinen, Linz, Austria; Department of Cardiology, University Hospital Basel, Basel, Switzerland; Cardiovascular Research Institute Basel, University Hospital Basel, Basel, Switzerland; Department for Cardiovascular Medicine, University Hospital Dubrava, Zagreb, Croatia; Department of Cardiology, Institute for Clinical and Experimental Medicine, Prague, Czech Republic; Department of Internal Medicine III (Cardiology and Intensive Care Medicine), University Hospital Schleswig-Holstein (UKSH), Kiel, Germany; Department of Cardiology, Inselspital, Bern University Hospital, University of Bern, Bern, Switzerland; Division of Cardiology, Department of Medicine, Medical University of Graz, Graz, Austria; Department of Internal Medicine, Cardiology and Nephrology, Hospital Wiener Neustadt, Wiener Neustadt, Austria; 1st Department of Cardiology, Interventional Electrocardiology, and Hypertension, Jagiellonian University Medical College, Krakow, Poland; 5th Department of Internal Medicine, Cardiology Rehabilitation, Iuliu Hatieganu University of Medicine and Pharmacy of Cluj Napoca, Cluj Napoca, Romania; Department of Cardiology, University Medical Centre of Ljubljana, Ljubljana, Slovenia; 1st Clinic of Cardiology and Electrotherapy, Swietokrzyskie Cardiology Centre, Kielce, Poland; Collegium Medicum, Jan Kochanowski University, Kielce, Poland; Cardioangiologisches Centrum Bethanien, Department Kardiologie, Markus Krankenhaus, Frankfurt, Germany; Department of Internal Medicine II, Cardiology, Angiology, and Intensive Care Medicine, Ordensklinikum Linz Elisabethinen, Linz, Austria; Department of Internal Medicine II, Cardiology, Angiology, and Intensive Care Medicine, Ordensklinikum Linz Elisabethinen, Linz, Austria; Department of Internal Medicine II, Cardiology, Angiology, and Intensive Care Medicine, Ordensklinikum Linz Elisabethinen, Linz, Austria; Department of Internal Medicine II, Cardiology, Angiology, and Intensive Care Medicine, Ordensklinikum Linz Elisabethinen, Linz, Austria; Cardioangiologisches Centrum Bethanien, Department Kardiologie, Markus Krankenhaus, Frankfurt, Germany; Heart Center, Department of Electrophysiology, University Hospital Cologne, Cologne, Germany; Heart Center, Department of Electrophysiology, University Hospital Cologne, Cologne, Germany; Clinic for Electrophysiology, Herz- und Diabeteszentrum NRW, Ruhr-Universitaet Bochum, Bad Oeynhausen, Germany; 1st Department of Cardiology, Interventional Electrocardiology, and Hypertension, Jagiellonian University Medical College, Krakow, Poland; Department of Cardiology, Institute for Clinical and Experimental Medicine, Prague, Czech Republic; Department of Cardiology, Inselspital, Bern University Hospital, University of Bern, Bern, Switzerland; Department of Cardiology, University Hospital Basel, Basel, Switzerland; Cardiovascular Research Institute Basel, University Hospital Basel, Basel, Switzerland; Department of Internal Medicine II, Cardiology, Angiology, and Intensive Care Medicine, Ordensklinikum Linz Elisabethinen, Linz, Austria; Clinical Electrophysiology, Hospital of the University of Pennsylvania, Philadelphia, Pennsylvania, USA; Heart Center, Department of Electrophysiology, University Hospital Cologne, Cologne, Germany; Cardioangiologisches Centrum Bethanien, Department Kardiologie, Markus Krankenhaus, Frankfurt, Germany

**Keywords:** Bipolar ablation, Ventricular tachycardia, Premature ventricular complexes, Advanced ablation strategies

## Abstract

**Aims:**

Advanced ablation strategies are needed to treat ventricular tachycardia (VT) and premature ventricular complexes (PVC) refractory to standard unipolar radiofrequency ablation (Uni-RFA). Bipolar radiofrequency catheter ablation (Bi-RFA) has emerged as a treatment option for refractory VT and PVC. Multicentre registry data on the use of Bi-RFA in the setting of refractory VT and PVC are lacking. The aim of this Bi-RFA registry is to determine its real-world safety, feasibility, and efficacy in patients with refractory VT/PVC.

**Methods and results:**

Consecutive patients undergoing Bi-RFA at 16 European centres for recurring VT/PVC after at least one standard Uni-RFA were included. Second ablation catheter was used instead of a dispersive patch and was positioned at the opposite site of the ablation target. Between March 2021 and August 2024, 91 patients underwent 94 Bi-RFA procedures (74 males, age 62 ± 13, and prior Uni-RFA range 1–8). Indications were recurrence of PVC (*n* = 56), VT (*n* = 20), electrical storm (*n* = 13), or PVC-triggered ventricular fibrillation (*n* = 2). Procedural time was 160 ± 73 min, Bi-RFA time 426 ± 286 s, and mean Uni-RFA time 819 ± 697 s. Elimination of clinical VT/PVC was achieved in 67 (74%) patients and suppression of VT/PVC in a further 10 (11%) patients. In the remaining 14 patients (15%), no effect on VT/PVC was observed. Three major complications occurred: coronary artery occlusion, atrioventricular block, and arteriovenous fistula. Follow-up lasted 7 ± 8 months. Nineteen patients (61%) remained VT free. ≥80% PVC burden reduction was achieved in 45 (78%).

**Conclusion:**

These real-world registry data indicate that Bi-RFA appears safe, is feasible, and is effective in the majority of patients with VT/PVC.

## Graphical Abstract

**Graphical Abstract:**
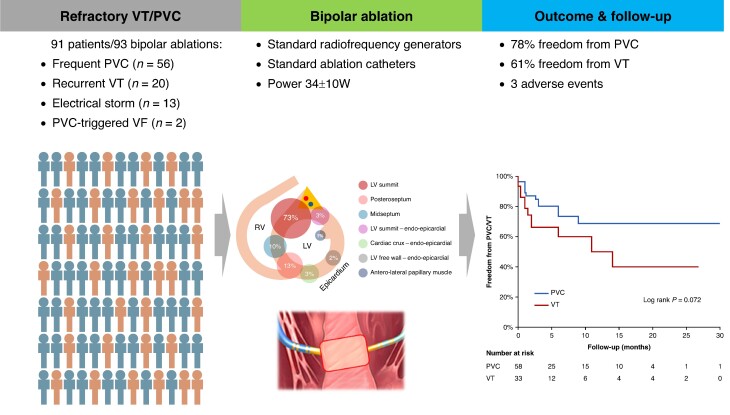

**What’s new?**

- This multicentre multinational registry provides detailed information on how bipolar radiofrequency catheter ablation can be used to treat arrhythmias in difficult anatomical areas, such as the interventricular septum and left ventricular summit, where traditional unipolar ablation is sometimes less effective.
- This study is the largest registry on bipolar ablation published to date, increasing the amount of data on its safety and efficacy.
- The registry highlights possible future directions for the ablation of challenging ventricular arrhythmias and identifies key areas that warrant further investigation, such as efficacy and safety in particular cardiac areas (i.e. left ventricular summit and interventricular septum) and clinical conditions (i.e. recurrent ventricular tachycardia in patients with heart failure).

## Introduction

Radiofrequency (RF) catheter ablation is an effective therapeutic strategy for idiopathic and scar-related ventricular arrhythmias (VAs).^1^ However, the efficacy of RF ablation can be limited when the arrhythmogenic substrate is intramural or epicardial (three-dimensional) and unipolar RF energy delivery might not be sufficient to thoroughly target the substrate or origin of VA.^2–4^

Bipolar RF catheter ablation (Bi-RFA) is an advanced ablation technique in which alternating current is applied between two ablation catheters placed on opposite sites of the target myocardium, with one of them connected to the active port of the RF generator [active catheter (AC)] and a second one connected to the indifferent port instead of the ground patch [return catheter (RC)]. Several case reports and clinical series have demonstrated that Bi-RFA can be effective in treating VAs that have been refractory to standard unipolar ablation.^5–12^ However, most of these studies have included small numbers of patients and have used custom-made equipment, with remaining concerns about the safety of this approach.^13,14^

The aim of this retrospective registry is to determine real-world safety, feasibility, and efficacy of Bi-RFA in consecutive patients with refractory ventricular tachycardia (VT) and premature ventricular complexes (PVCs).

## Methods

### Patients

This multicentre study included consecutive patients undergoing Bi-RFA at 16 European centres between March 2021 and August 2024. Inclusion criteria were age ≥ 18 years and refractory VT/PVC after at least one standard unipolar ablation attempt. Data acquisition and manuscript writing were promoted by the European Heart Rhythm Association (EHRA) scientific initiative committee network.

The study and collection of data were approved by the ethics committee and the Institutional Review Boards of the participating centres. All patients provided written informed consent prior to the ablation procedure. The baseline clinical characteristics including age, sex, clinical presentation, and 12-lead electrocardiogram (ECG) of the PVC/VT were recorded.

### Mapping

All procedures were conducted under conscious sedation or general anaesthesia. Whenever possible, antiarrhythmic medications were discontinued for five half-lives before the procedure. Oral anticoagulation was set individually according to each centre’s practice for VA ablation. Mapping was guided by electroanatomic mapping using the CARTO™ (Biosense Webster, Diamond Bar, CA, USA), Ensite™ (Abbott, St Paul, MN, USA), Rhythmia™ (Boston Scientific, Natick, MA, USA), or EP Navigator (Phillips, Best, the Netherlands) systems. For left ventricular (LV) mapping, a retrograde aortic and/or trans-septal approach was used and heparin was administered to maintain an activated clotting time of >300 s. Direct epicardial access technique using a ‘Tuohy’ or conventional needle and fluoroscopic guidance was used for mapping purposes within the pericardial space when needed.

For PVCs, activation mapping was used, detecting the earliest bipolar activation time compared with surface QRS onset. In case of infrequent PVCs, induction was attempted with isoproterenol infusion (2–20 mcg/min) or salbutamol boluses (0.125 mg) and ventricular/atrial burst pacing. Pace mapping was also used as a complement to activation mapping, and matching between the paced beats and the clinical PVCs was assessed using the PASO™ (CARTO™, Biosense Webster) and Score Map (Ensite™, Abbott) software modules.

For scar-related VT, the circuit was characterized using activation and entrainment mapping if haemodynamically tolerated. Sites with concealed QRS fusion and return cycle within 30 ms of the VT cycle length with matching stimulus-QRS and electrogram-QRS intervals or where VT terminated during pacing without global capture were considered critical. For haemodynamically unstable VTs, substrate modification was performed, targeting sites identified by pace mapping (similar QRS morphology and long stimulus to QRS interval), abnormal electrograms, and sites of isochronal crowding on isochronal late activation mapping.

### Bipolar ablation

All procedures were performed using standard RF generators—Ampere™ (St. Jude Medical, St. Paul, MN, USA), Smartablate™ (Biosense Webster, Irvine, CA, USA), nGen™ (Biosense Webster, Irvine, CA, USA), or EP Shuttle (Stockert, Freiburg, Germany). Two open-irrigated ablation catheters (Thermocool™, Themocool SF™, SmartTouch™, SmartTouch SF™, or QDot™, Biosense Webster, Diamond Bar, USA; Flexibility, Coolflex™, Tacticath™, or Tactiflex, Abbott, St. Paul, MN, USA) or non-irrigated 8 mm ablation catheters (Celsius™, Biosense Webster, Diamond Bar, USA; Therapy™, Abbott, St. Paul, MN, USA; Triguy™, APT, Shenzen, China) were used as AC and RC. The RC was connected to the indifferent port of the RF generator using a dedicated Dr Futyma Bipolar Ablation Adapter™ (CorSystem, Rzeszow, Poland). When one of the ablation catheters was in the coronary venous system or pericardial space, a coronary angiography was performed to confirm a safe distance (>5 mm) of the catheter tip from epicardial coronary arteries. The distance between ablation catheters was assessed using fluoroscopy and/or three-dimensional mapping system. The choice of locations for AC and RC was arbitrary and not strictly regulated by the protocol.

Radiofrequency ablation was performed by starting with lower power of 20–30 W and 10–20 W if one of the ablation catheters was positioned in the coronary veins and titrating up gradually to achieve an impedance drop of at least 10% from baseline values. For non-irrigated catheters, a temperature limit of 60°C was set. For standard open-irrigated catheters, temperature limit was set at 42°C, and for open-irrigated catheters equipped with multiple thermocouples, the temperature limit was set at 50°C. Irrigation rates of open-irrigated catheters were programmed according to the manufacturer’s recommendations.

### Outcomes and follow-up

Acute procedural success was defined as follows: (i) for PVC: complete elimination of the clinical spontaneous or inducible PVC after a waiting time of 30 min, despite isoproterenol or salbutamol administration, wake up manoeuvres and repeat deep sedation in case of sedated patients; and (ii) for VT: non-inducibility of any sustained monomorphic VT at the end of the procedure. Serious complications were defined as those that caused prolonged hospitalization or resulted in significant morbidity or mortality. These included pericardial effusion with tamponade, transient or permanent cerebrovascular event, complete heart block, acute coronary syndrome, major bleeding requiring transfusion, or vascular injury that required surgical intervention.

After the procedure, patients remained in the hospital overnight under continuous ECG monitoring and were discharged the next day if no complications occurred. Follow-up included inpatient telemetry, outpatient rhythm monitoring, device interrogation if applicable, and follow-up clinic visits. In PVC patients, a 24- to 72-h Holter or a 2-week event monitor was obtained to assess residual PVC burden between 1 and 3 months after discharge. Follow-ups were performed at intervals deemed necessary by the treating physician or as clinically indicated.

### Statistical analysis

Results were summarized as mean ± standard deviation for continuous variables and as frequency and percentage for categorical variables. For continuous variables, analyses were performed using two-tailed Student’s *t*-tests. For categorical variables, *χ*^2^ or Fisher's exact tests were used, with Yates’ correction applied when appropriate. Kaplan–Meier analysis was conducted to determine the recurrence rate of VT/PVC and its timing. A *P*-value < 0.05 was considered statistically significant.

## Results

Between March 2021 and August 2024, 91 consecutive patients underwent a total of 94 Bi-RFA procedures (74 males, age 62 ± 13, body mass index 28.8 ± 4.6, number of prior ablations attempts 1.6 ± 1.2; range 1–8). The main indications for ablation were frequent PVCs (*n* = 56), monomorphic VT (*n* = 20), electrical storm (*n* = 13), or PVC-triggered ventricular fibrillation (*n* = 2) reoccurring after standard Uni-RFA. Clinical and demographic characteristics of the study population are presented in *Table 1*. Patients with VT had a lower LV ejection fraction (LVEF; 41 ± 13% vs. 50 ± 11%, *P* = 0.0008), a higher prevalence of prior myocardial infarction (24% vs. 5%, *P* = 0.02), and more frequent prior percutaneous coronary intervention (30% vs. 10%, *P* = 0.02). Additionally, hypertrophic cardiomyopathy was present in 9% of VT patients compared with 0% of those with PVC (*P* = 0.045). Moreover, patients with VT were more likely to take β-blockers (91% vs. 71%, *P* = 0.03) and amiodarone (58% vs. 12%, *P* = 0.00001).

**Table 1:** Comparison of clinical characteristics in the PVC/PVC triggered VF and VT/VT storm groups

	PVC/PVC-triggered VF *n* = 58	VT/VT storm *n* = 33	*P*-value
Males	44 (76%)	30 (91%)	0.08
Age (years)	60 ± 13	65 ± 11	0.09
Number of previously failed ablations	1.4 ± 1.1	1.9 ± 1.4	0.12
LVEF (%)	50 ± 11	41 ± 13	0.008
Hypertension	36 (62%)	21 (64%)	0.94
Diabetes	8 (14%)	8 (24%)	0.21
Prior myocardial infarction	3 (5%)	8 (24%)	0.02
Prior coronary artery bypass grafting (CABG)	3 (5%)	2 (6%)	0.76
Prior percutaneous coronary intervention (PCI)	6 (10%)	10 (30%)	0.03
Valvular disease	3 (5%)	6 (18%)	0.09
β -blockers use	41 (70%)	30 (91%)	0.03
Amiodarone use	7 (12%)	19 (58%)	0.00001
IC class use	10 (17%)	7 (21%)	0.64
Ischaemic aetiology	5 (9%)	7 (21%)	0.09
Idiopathic aetiology	34 (59%)	7 (21%)	0.0006
Non-ischaemic aetiology	19 (33%)	19 (58%)	0.02
Hypertrophic cardiomyopathy	0 (0%)	3 (9%)	0.045
Dilated cardiomyopathy	9 (16%)	12 (36%)	0.02
Post-myocarditis cardiomyopathy	2 (3%)	2 (6%)	0.96
Unspecified non-ischaemic cardiomyopathy	4 (7%)	1 (3%)	0.76
Tachycardia-induced cardiomyopathy	2 (3%)	1 (3%)	0.61
Left ventricular non-compaction cardiomyopathy	2 (3%)	0 (0%)	0.53

LVEF, left ventricular ejection fraction; PVC, premature ventricular complex; VF, ventricular fibrillation; VT, ventricular tachycardia.

**Figure 1:**
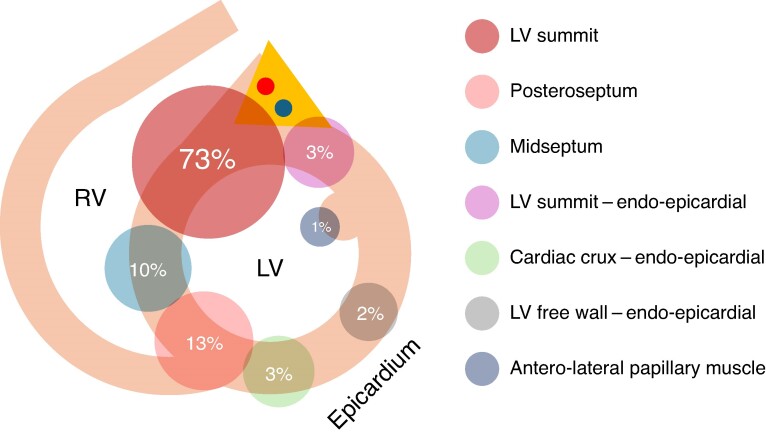
Schematic distribution (%) of Bi-RFA lesion sets in patients included in the study. LV, left ventricle; RV, right ventricle.

**Figure 2:**
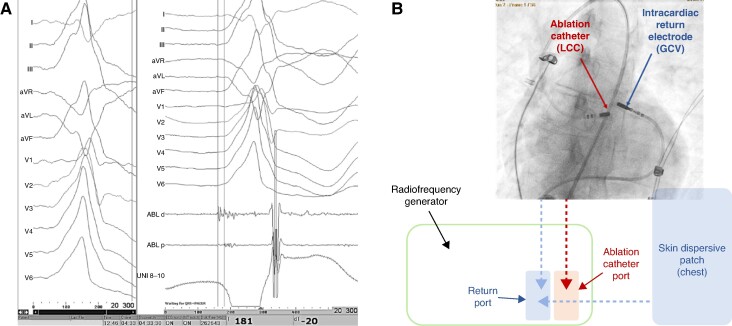
(*A*) Clinical PVC of a 56-year-old female after two failed extensive unipolar ablation attempts. Earliest activation preceding PVC onset by −20 ms was recorded in the great cardiac vein adjacent to the LV summit area. (*B*) Configuration of the catheters and their connection to the RF generator during bipolar ablation with addition of dispersive patch to deal with the return electrodes overheating of the intracardiac return electrode positioned in the GCV. In such setting, the RF current alternates between ablation catheter tip located in the LCC and both: tip of the 8 mm intracardiac return electrode in GCV and skin dispersive patch located at the anterior chest, which anatomically is the closest surface area to the LV summit region. Power was up titrated to 40 W with effective PVC elimination and no recurrence in the follow-up. Tip temperature of the GCV catheter in such configuration did not exceed 60°C. GVC, great cardiac vein; LCC, left coronary cusp.

In 79 procedures, a combined unipolar and bipolar ablation approach was used, while only bipolar ablation was used in the remaining 15 cases. Intraprocedural data for PVC and VT procedures are compared in *Table 2*. The mean procedural time for VT procedures was significantly longer than for PVC procedures (188 ± 85 min vs. 144 ± 59 min, *P* = 0.004). Additionally, the mean time for bipolar applications was significantly shorter compared with unipolar applications (426 ± 286 s vs. 819 ± 697 s, *P* < 0.0001), energy used for bipolar RF applications was significantly lower than that for unipolar applications (34 ± 10 W vs. 45 ± 9 W, *P* < 0.0001), and the number of bipolar applications was significantly lower that the number of unipolar applications (8 ± 6 vs. 17 ± 18, *P* < 0.0001). Values of power and duration of unipolar applications reflect only the combined Uni-RFA and Bi-RFA procedures and do not include cumulative values from previous procedures. Most procedures were performed under deep sedation (95%). General anaesthesia was used in three cases (3%), and two cases (2%) were performed without any sedation. In 64 procedures (68%), access to the LV was obtained via retrograde aortic approach, while trans-septal puncture was performed in 21 cases (22%). In nine patients (10%), both approaches were used. The LV summit area was the primary ablation target in 67 (74%) patients. For the LV summit region, Bi-RFA ablation was attempted between distal cardiac veins and the LV outflow tract (LVOT) in 26 patients (lateral LV summit region), between left pulmonic cusp and LVOT in 8 patients (inaccessible LV summit region), and between right ventricular outflow tract (RVOT) and LVOT in 33 patients (anteromedial LV summit region). Of these patients, 14 (16%) required sequential Bi-RFA using multiple of the above-listed bipolar configurations. In 21 (23%) patients, Bi-RFA of VT/PVC originating from the intraventricular septum (IVS) was performed, with 12 (13%) of these cases originating from the posteroseptal region and 9 (10%) from the mid-septal region. In one patient (1%), a focus of PVC was targeted in the anterolateral papillary muscle with both ablation catheters introduced to the LV cavity to the targeted area. In another patient, two morphologies of VT were present, both targeted with Bi-RFA—in posteroseptal region of IVS and in the anteromedial LV summit. Epicardial ablation with sub-xiphoid puncture to access the epicardium was performed in eight (9%) patients. Detailed distribution of Bi-RFA lesion sets is depicted in the *Figure 1*. Overall, Bi-RFA was feasible in 88 patients (97%). In the remaining three (3%) patients, all with LV summit VT/PVC and with one of two ablation catheters positioned in the distal coronary veins, a significant overheating of the catheter positioned within the cardiac veins was observed, which required termination of bipolar applications. This issue was managed with energy down-titration or by the addition of a dispersive patch to the anterior chest^15^ to the existing bipolar configuration (*Figure 2*). Acute elimination of the clinical VT/PVC was achieved in 67 (74%) patients, while significant suppression of clinical VT/PVC was observed in 10 (11%) patients. In the remaining 14 patients (15%), no effect on clinical arrhythmia was observed. Concomitant alcohol ablation was performed in three patients and in one patient as a redo ablation.

**Table 2:** Comparison of intraprocedural data in the PVC/PVC triggered VF and VT/VT storm groups

	PVC/PVC-triggered VF *n* = 59	VT/VT storm *n* = 35	*P*-value
Time of bipolar applications (s)	384 ± 238	504 ± 344	0.06
Power of bipolar applications (W)	33 ± 11	36 ± 7	0.28
Time of unipolar applications (s)	706 ± 521	1051 ± 918	0.053
Power of unipolar applications (W)	44 ± 10	48 ± 7	0.12
Epicardial access	1 (2%)	7 (20%)	0.006
Intracardiac echocardiography	5 (9%)	3 (9%)	0.76
General anaesthesia	1 (2%)	2 (6%)	0.65
Trans-septal access	6 (10%)	15 (43%)	0.0001
Retroaortic access	51 (88%)	13 (37%)	<0.00001
Trans-septal and retroaortic access	2 (3%)	7 (20%)	0.02
Carto™ mapping system	23 (39%)	18 (51%)	0.24
Ensite™ mapping system	21 (36%)	12 (34%)	0.90
EP Navigator™ mapping system	14 (24%)	4 (11%)	0.14
Rhythmia™ mapping system	1 (2%)	1 (3%)	0.72
Procedure time (min)	144 ± 59	188 ± 85	0.004

PVC, premature ventricular complexes; VF, ventricular fibrillation; VT, ventricular tachycardia.

**Figure 3:**
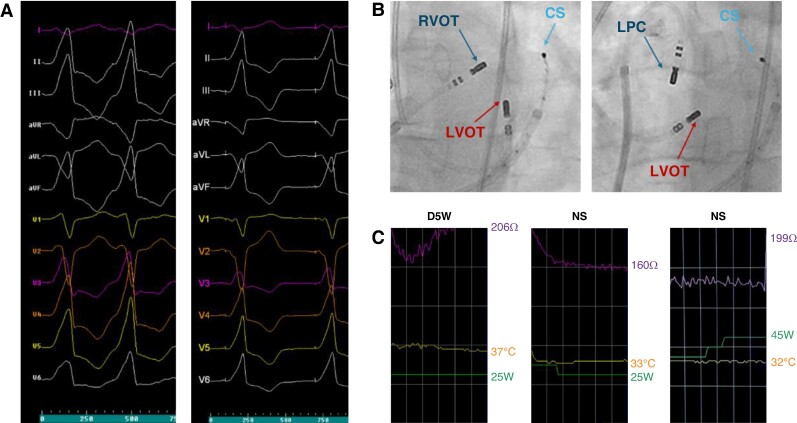
(*A*) Clinical VT of a 39-year-old male after eight failed Uni-RFA attempts and one Bi-RFA attempt. Abrupt V3 transition pattern is suggestive for LV summit origin. During repeat Bi-RFA ablation best, although imperfect, pace mapping was found at the septal region of the LVOT area. (*B*) Sequential Bi-RFA preformed between the LPC, RVOT, and LVOT with the use of two flexible-tip (Flexibility, Abbott, USA) ablation catheters. (*C*) Impedance trends during Bi-RFA observed with D5W and NS irrigation of both ablation catheters and at different power settings. CS, coronary sinus; D5W, dextrose-5-in water; LPC, left pulmonic cusp; LVOT, left ventricular outflow tract; NS, normal saline; RVOT, right ventricular outflow tract.

Three patients required repeat Bi-RFA after the initial procedures failed. The first patient with VT and issues with baseline VT inducibility during the initial procedure underwent anatomical high-power bipolar ablation targeting the anteroseptal region of the LV summit. Given the initial non-inducibility issue, the repeat procedure was performed until complete non-excitability of the targeted LV summit area was achieved from both sites using high output pacing (10 mA, 2 ms), and no further VT recurrence was observed during follow-up (*Figure 3*). In the second patient, due to poor stability of the ablation catheters during the initial Bi-RFA, the repeat ablation was conducted using two contact force-sensing ablation catheters connected to two separate contact force-sensing modules. No VT recurrence was observed during follow-up. A third patient experienced recurrence of PVCs 1 month after the initial procedure and underwent repeat ablation. The arrhythmia recurred after the second procedure; however, the patient experienced significant improvement of symptoms.

### Complications

There were three (3%) major complications. One case involved occlusion of the left anterior descending artery (LAD) during Bi-RFA applications between the epicardial LV and the adjacent LVOT. Coronary angiogram preceding bipolar applications was performed. The RF delivery was started 2 cm from the LAD at the site of optimal pace mapping. During the last bipolar application, the catheter dislodged, and a sudden drop in mean arterial pressure and ST-elevations in leads I, aVL, and V2–5 was observed. Immediate coronary angiography revealed >90% stenosis of the LAD without improvement after intracoronary nitroglycerine administration. Percutaneous angioplasty was subsequently performed, which restored normal coronary flow. During follow-up, the patient recovered and the LVEF remained unchanged during follow-up. One anticipated atrioventricular (AV) block occurred during extensive Bi-RFA application at a mid-ventricular septal area in a patient already equipped with a cardiac resynchronization therapy device; thus, no additional therapeutic intervention was required. Finally, one arteriovenous fistula occurred in one patient. This was diagnosed the day after the procedure and was managed conservatively. After 20 days, the fistula could not be longer visualized on ultrasonography.

Minor complications included one asymptomatic coronary vein dissection, which occurred during advancement of a flexible-tip ablation catheter to the great cardiac vein (GCV). Follow-up echocardiography revealed a 5 mm pericardial effusion without symptoms, and no therapeutic intervention was needed. Also, one char formation on non-irrigated catheter tip positioned in the GCV, without any sequelae, was observed.

No post-procedural ventricular septal defect (VSD) after Bi-RFA was detected, but more long-term data are needed.

### Follow-up

Follow-up lasted 7 ± 8 months. Shortly after the procedure, two patients died. The first one was a patient with advanced-stage heart failure and reduced EF (25%) that presented with VT storm, and he died due to severe hyperkalaemia 1 week after ablation. The second patient, with VT storm, previous coronary artery bypass grafting, and heart failure (EF = 17%), developed pulseless electrical activity 2 days after the procedure and was treated with extracorporeal membrane oxygenation but died 4 days later.

Nineteen (61%) out of the remaining 31 VT patients remained VT free during follow-up, including 7 (37%) on antiarrhythmic drugs (5 patients received amiodarone, one was treated with amiodarone and mexiletine, and one received sotalol). While a proportion of patients experienced VT recurrences during follow-up, a possible improvement of VT burden was not assessed in the current analysis.

**Figure 4:**
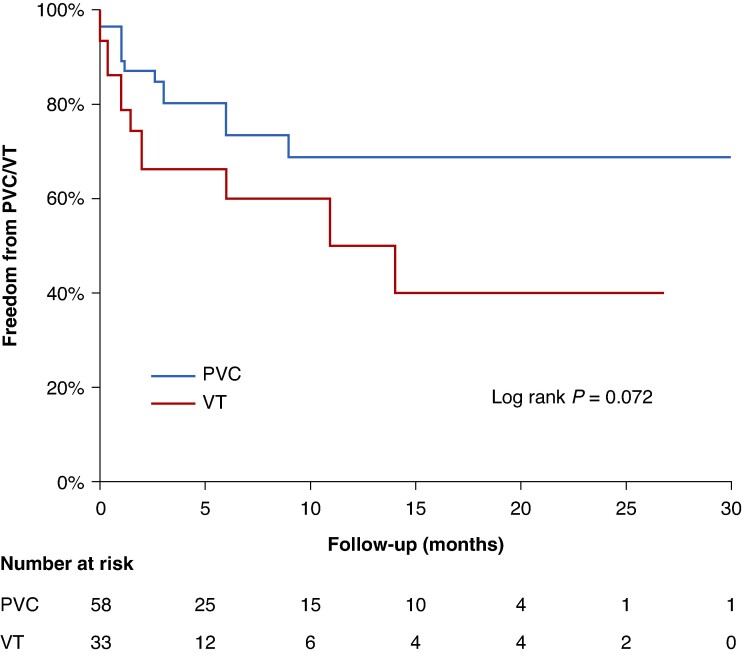
Kaplan–Meier curves demonstrating freedom from the clinical PVCs and VT after Bi-RFA. PVC, premature ventricular complexes; VT, ventricular tachycardia.

Significant PVC burden reduction, defined as ≥80% PVC burden reduction, was achieved in 45 (78%) out of 58 PVC patients during follow-up, including 5 (11%) on antiarrhythmic drugs (2 patients received flecainide, 2 were treated with sotalol, and 1 received amiodarone) (*Figure 4*).

Apart of the two patients who died within 1 week after the procedure, three more patients died during extended follow-up. A 75-year-old male with VT storm and septal substrate died 4 months after the ablation due to severe pneumonia and septic shock. The second patient, a 72-year-old male with ischaemic VT storm, suffered from a recurrence of VT 6 weeks after the Bi-RFA. During redo ablation, seven different morphologies of VT were found. This patient died 6 months after the first procedure due to cardiogenic shock and concomitant sepsis in the course of Clostridium difficile and Covid-19 infection. The third patient, a 79-year-old male with dilated cardiomyopathy and VT storm, had VT storm recurrence immediately after the Bi-RFA procedure. During redo ablation, he was treated with stereotactic radiotherapy but died during follow-up.

## Discussion

This multinational multicentre retrospective study demonstrates that bipolar ablation can achieve success in many patients with VT/PVCs who have failed standard Uni-RFA and is associated with a relatively low rate of complications related directly to Bi-RFA. However, recent data reveal that this ablation modality is underutilized.^13^ To the best of our knowledge, this is the largest series of patients treated with bipolar ablation and provides a reproducible workflow that uses common commercially available ablation catheters, standard RF generators, and a dedicated bipolar connection.

### Challenges in ventricular arrhythmias ablation

During RF ablation of Vas, certain challenges might hamper the complete treatment of the underlying three-dimensional substrate or origin. Ventricular tachycardia and PVCs often originate from deep intramural sites or epicardial regions, which are difficult to reach with standard Uni-RFA.^3^ A particularly challenging for Uni-RFA region—the LV summit—is a triangular area situated at the most superior part of the LV epicardium. This area is bordered by the two branches of the left coronary artery: the left anterior interventricular artery and the left circumflex artery. Due to inaccessibility, proximity to major structures such as the coronary arteries, a significant epicardial fat layer, and the fibrotic components of the aortic and pulmonic valves, ablation in this region is particularly difficult.^16–19^ Additionally, the thickness of certain regions, such as the IVS, may further complicate the ablation. The presence of adipose and scar tissue, common in patients with structural heart disease or prior myocardial infarction,^20,21^ may impede the penetration of RF energy and limit lesion depth. The variable resistivity within scarred tissue leads to uneven energy absorption and non-uniform tissue injury.^22^ Consequently, achieving consistent lesion formation and transmural penetration becomes difficult with a standard Uni-RFA approach.^23,24^

In our study, despite the use of relatively high powers (45 ± 9 W) and long durations of applications (819 ± 697 s), Uni-RFA did not lead to the effective elimination of arrhythmias. Conversely, the lower power required for Bi-RFA lesions likely reflects the recognition of bipolar mode as more efficient for intramural substrates, as suggested by previous *ex vivo* and *in vivo* studies.^24–26^ However, the actual limit of Uni-RF applications remains undetermined, especially given the fact that some complications following extensive Uni-RFA have already been reported on multiple occasions in the literature so far.^27–30^ Additionally, extensive high power ablation does not seem to benefit much if extended above 90 s, according to pre-clinical model.^31^ It is important to acknowledge that a situation where an extensive Uni-RFA induces significant oedema, leading to increased tissue thickness, may occasionally occur and limit the efficacy of subsequent Bi-RFA lesions.^32^ However, some studies observed an opposite effect—with the repeated RF applications, the lesion size progressively increased, suggesting the role of tissue preconditioning with RF current.^33^ Although the initial lesion may not reach maximum depth, it reduces tissue impedance, enabling deeper penetration during subsequent RF applications.^33^

Another anatomical factor that makes conventional ablation difficult is the heat sink effect. This phenomenon is particularly significant in proximity to major vessels like the aorta or coronary arteries and veins. With dynamic blood flow from the LV acting as an efficient coolant, the rapid dissipation of thermal energy significantly limits temperature elevation within the targeted tissue.^34^ This cooling effect poses substantial difficulty in creating effective lesions during RF application. Classic Uni-RFA may be particularly susceptible to the heat sink effect, leading to suboptimal lesion formation, especially in scar-related VT.^34^ In contrast, Bi-RFA may offer mitigation of the heat sink effect by providing more concentrated and controlled energy delivery between bipolar electrodes. The amount of alternating current is the same for each tip of two ablation catheters connected within the Bi-RFA circuit; however, some discrepancies may be observed depending on the tip size and tissue characteristics. Previous *ex vivo* and *in vivo* studies showed that the use of 8 mm in parallel orientation can be beneficial for the optimization of Bi-RFA lesions in the GCV.^7,35^ Moreover, 8 mm catheter may be less prone for overheating.^9,14^ Thus, the use of large tip RCs can be particularly beneficial when positioned in the venous system for LV summit VT/PVC^6^ or right ventricular side of the IVS.

Furthermore, the frequently observed presence of multiple early activation sites of intramural VAs further complicates its precise endocardial mapping. Recent evidence suggests that in cases where activation mapping identifies more than one early activation site, sequential ablation at each site is necessary for long-term success.^36^ Ablation targeting only a single site, even one with the earliest activation, often results in incomplete suppression, as these arrhythmias frequently have a deep, intramural origin. When bipolar ablation is performed, the vector of RF current flow can be altered, which can extend the ablated area. For Bi-RFA, various electrode configurations across multiple sites are sometimes applied, further increasing the lesion dimensions.^37^

Certain techniques, such as prolonged RF applications, higher contact force, half normal saline irrigation, epicardial ablation, or needle-tipped ablation catheter, may improve procedural outcomes.^26,38–40^ The use of dedicated adapter for standard RF generators can provide simple and standardized solution for the enhancement of RF current delivery, making such advanced ablation strategy more available in electrophysiology centres.^13^

### Differences between ventricular tachycardia and premature ventricular complexes outcomes

The outcomes of PVC and VT ablation differ significantly, mainly due to the underlying pathophysiology and clinical characteristics associated with these arrhythmias. Ventricular tachycardia ablation outcomes tend to be less favourable compared with PVC ablation (in our study 61% vs. 78% freedom from recurrences), primarily due to the higher prevalence of structural heart disease among VT patients.^41,42^ Structural heart disease, such as ischaemic cardiomyopathy or non-ischaemic dilated cardiomyopathy, is a common substrate for VT, leading to a more complex arrhythmogenic substrate and increased number of procedural difficulties.^13,43^ Additionally, the progression of substrate complexity in VT patients, characterized by scar formation, fibrosis, and myocardial remodelling, further complicates ablation procedures and contributes to higher rates of arrhythmia recurrence. Ventricular tachycardia ablation is also associated with a higher risk of adverse outcomes, including mortality, due to the underlying structural heart disease and the potential for haemodynamic compromise during sustained VT episodes.^42^ In contrast, PVC ablation outcomes tend to be more favourable, as PVCs are often idiopathic and not associated with significant structural heart disease.

### Complications of bipolar ablation

Complications associated with bipolar ablation, although rare, can have significant clinical implications.^44^ While our study demonstrates a low incidence of complications, it is crucial to understand potential adverse events to ensure procedural safety. Apart from anticipated complications such as conduction disturbances, including AV block,^45,46^ other potential issues may arise during or after bipolar ablation procedures.

An especially significant concern during bipolar ablation procedures is the risk of thermal injury to the coronary arteries. The proximity of the LV summit to the coronary vessels poses a potential risk of coronary artery injury, which can lead to occlusion of the coronary artery and myocardial infarction.^46^ Keeping the safe distance > 5 mm from the left anterior descending artery (LAD) is crucial for safe Bi-RFA applications.^6,18^ Such distance is extrapolated from previous studies dealing with unipolar ablation performed in near proximity of coronary arteries^47–49^ and recent Heart Rhythm Society (HRS)/EHRA/Asia-Pacific Heart Rhythm Society (APHRS)/Latin American Heart Rhythm Society (LAHRS) expert consensus statement on catheter ablation of VAs,^1^ but more data for bipolar ablation are needed.

Another concern is the development of pericardial effusion or tamponade, especially during septal or epicardial ablation procedures, where the pericardium is at risk of injury.^50^ Careful attention to catheter positioning and real-time monitoring of the catheter temperature and impedance can mitigate this risk.

No VSD was observed in our study. This rare complication was reported as an adverse event of Bi-RFCA in one recent case series.^51^ Ventricular septal defect, however, has not been described in any of the previously published studies on bipolar ablation.^25,46,50^ Also, no VSD was reported in the bipolar VT study, according to the ClinicalTrials.gov.^52^ However, it should be acknowledged that although none of our study patients developed VSD during the follow-up, such defects may occur after a relatively long time period after Bi-RFA.^51^ Thus, future studies on Bi-RFA should include longer follow-up screening for any possible VSD.

Two patients with VT storm and decompensated heart failure died after a few days following Bi-RFA. It is important to acknowledge that in patients with severely impaired EF and recurrent malignant VTs, ablation procedure is associated with substantial peri-procedural risks, particularly acute haemodynamic decompensation (AHD), which correlates strongly with short-term mortality.^53^ The PAINESD risk score serves as a useful predictor for AHD and adverse post-procedural outcomes, assisting in identifying patients who may benefit from pre-procedural haemodynamic optimization and mechanical support. Recent data indicated a high mortality rate among patients with advanced HF and VT storm experiencing AHD during catheter ablation, with a substantial number of deaths occurring during the same hospital stay.^53^

### Other bipolar multicentre studies

Results from our study are mostly consistent with previous multicentre studies that included smaller number of patients.^25,46,50,52^ These studies also confirm that Bi-RFA can be an effective strategy for treating refractory VAs, particularly when traditional Uni-RFA methods have failed. Acute success rates with Bi-RFA were notably high. One study reported success in all cases of VT and a significant proportion of PVCs.^25^ Another two studies also confirmed a high rate of acute success, achieving VT suppression in 89% and 95% of cases, respectively.^46,50^ Although Bi-RFA presents high percentage of acute success, recurrence rates remain substantial. In our study, the recurrence rate of VT was 39%, which aligns with the recurrence rates reported in other studies, ranging from 33% to 45%.^25,52^ However, the effectiveness of PVC ablation during follow-up was higher in our study (78%) compared with previous reports where it was 50%.^25^ Complications associated with Bi-RFA, such as complete AV block, coronary artery stenosis, or tamponade, were relatively infrequent. In one study, a single case of complete AV block was noted^29^; in another, tamponade occurred^50^; and in a third, two cases of AV block and one case of coronary artery occlusion were documented.^46^ The higher rate of procedural complications compared with standard Uni-RFA approaches indicates that Bi-RFA may be associated with a greater risk, particularly in complex cases. It should be acknowledged that, in the setting of diffuse mid-septal substrate for VT, extensive Bi-RFA may be necessary to achieve non-inducibility and, in such circumstances, a complete AV block may be actually anticipated and possible device implantation or upgrade should be discussed with the patient before Bi-RFA.^9^

### Evolution of ventricular arrhythmia ablation and future directions

In recent years, ablation of VAs has evolved significantly. While unipolar RF ablation remains the primary method, new technologies like Bi-RFA, stereotactic body radiation therapy, and ethanol ablation have significantly improved efficacy, particularly in challenging regions like the IVS and LV summit.^54^ Substrate mapping and high-density mapping techniques have improved accuracy, enabling better identification of arrhythmogenic regions. Additionally, advancements in imaging, such as intracardiac echocardiography, late gadolinium enhancement magnetic cardiac resonance, and computed tomography, have facilitated more precise ablation targeting.^55^

Future directions in the treatment of intramural VAs are focused on improving outcomes and ensuring safety. There is a necessity for prospective registries with patient long-term outcomes and procedural variables. Future studies on Bi-RFA should be more standardized, including patients with a more homogeneous aetiology of VAs, pre-defined ablation targets, and appropriate control groups. Moreover, these studies should use more uniform equipment and power settings to ensure consistency across procedures. Ideally, randomized controlled trials on homogenous cohorts of patients with VT/PVC, comparing Bi-RFA with antiarrhythmic drugs or other ablation modalities, such as alcohol ablation^56^ or ultra-low cryoablation,^57^ should be warranted.

Bipolar ablation is not the only treatment method for intramural VAs. Pulsed field ablation (PFA) offers the precise, non-thermal ablation that selectively targets cardiac tissue while sparing surrounding structures. Recent studies have shown high effectiveness of PFA in other cardiac arrhythmias such as atrial fibrillation.^58^ However, only a few case studies of VA using PFA have been described, demonstrating promising results.^59–61^ Recent paper on larger series documented fair acute and long-term success after PFA with a solid tip technology, especially for PVC, which failed previous Uni-RFA.^62^ Further research is needed to fully specify its efficacy and safety profile.

### Study limitations

This is a retrospective study. All patients, at some point, received both unipolar and bipolar ablations, so the results of the current study may demonstrate the cumulative effect of both ablation modalities. Although the tools utilized were similar, the decision on when to use bipolar ablation and the ablation settings was not uniform. As a single-arm registry, this study lacks a control group, making it difficult to directly compare Bi-RFA outcomes with those of standard Uni-RFA or other advanced ablation techniques. Moreover, the follow-up is relatively short, which may overestimate the success rate.

## Conclusion

In most patients with PVC/VT refractory to standard Uni-RFA, an approach enhanced with Bi-RFA demonstrates high acute efficacy and an acceptable safety profile. Future research should focus on validating these results in larger, prospective, controlled trials and on comparing Bi-RFA with other novel ablation techniques.

## Data Availability

The data underlying this article will be shared on reasonable request to the corresponding author.
